# The interaction between illness perception and intrinsic capacity during hospital-to-home transition in older adults with COPD and diabetes: a prospective cohort study

**DOI:** 10.3389/fmed.2026.1896128

**Published:** 2026-07-22

**Authors:** Xinjing Peng, Xiaoqing Cheng, Haijuan Shen, Qin Xiao

**Affiliations:** 1 Department of Tuberculosis, The Sixth People's Hospital of Nantong, Nantong, Jiangsu, China; 2 Department of Respiratory Medicine, The Sixth People's Hospital of Nantong, Nantong, Jiangsu, China; 3 Department of Pulmonary Disease and Intensive Care Medicine, Nanjing Gaochun People's Hospital, Nanjing, Jiangsu, China

**Keywords:** chronic obstructive pulmonary disease, illness perception, intrinsic capacity, longitudinal studies, older adults, patient transition, type 2 diabetes mellitus

## Abstract

**Objective:**

To investigate the changes, bidirectional relationships, and moderating factors of illness perception and intrinsic capacity in older adults with COPD and Type 2 Diabetes Mellitus (T2DM) during the hospital-to-home transition.

**Methods:**

A prospective cohort study was conducted from April 2024 to September 2025, conveniently enrolling 232 older adults with COPD and T2DM from the Sixth People’s Hospital of Nantong. Participants were assessed at three time points: at discharge (T0), 1 month (T1), and 3 months (T2) post-discharge, using the Brief Illness Perception Questionnaire (BIPQ) and a WHO framework-based intrinsic capacity assessment tool. A Random-intercept cross-lagged panel model (RI-CLPM) was used to analyze the dynamic cross-lagged relationships, a Latent growth model (LGM) was used to analyze the association of their change trajectories, and exploratory subgroup analyses were performed.

**Results:**

(1) Trajectory analysis: Illness perception total scores showed a consistent and significant decreasing trend across the three time points (*p* < 0.001), while intrinsic capacity total scores exhibited a slight but significant linear decline (*p* = 0.010). (2) Dynamic associations: The RI-CLPM revealed significant bidirectional negative associations between illness perception and intrinsic capacity during the T0-to-T1 phase (*β* = −0.21, *β* = 0.18, both *p* < 0.05). During the T1-to-T2 phase, only intrinsic capacity was positively associated with subsequent higher negative illness perception (*β* = 0.15, *p* = 0.047). (3) Trajectory association: The parallel process LGM indicated a moderate-to-strong positive correlation between the rate of improvement in illness perception and the rate of decline in intrinsic capacity (*r* = 0.32, *p* = 0.008). (4) Subgroup differences: Exploratory analyses showed that COPD severity (GOLD stage 3–4) and impaired baseline vitality significantly strengthened the negative association between illness perception and intrinsic capacity (between-group comparison *p* < 0.05).

**Conclusion:**

During the hospital-to-home transition, illness perception and intrinsic capacity in older adults with COPD and T2DM exhibit a dynamic interaction, characterized by bidirectional influences in the early stage and a more sustained effect of intrinsic capacity on perception later. COPD severity and baseline vitality status are important clinical moderators of this relationship. The findings provide a basis for developing targeted transition care strategies that integrate psychological and functional interventions for high-risk subgroups.

## 1 Introduction

Chronic Obstructive Pulmonary Disease (COPD) and diabetes mellitus are a common comorbid combination. They mutually exacerbate each other, leading to a heavy symptom burden and increased risk of re-hospitalization for patients, presenting a significant global public health challenge (1, 2). With the accelerating aging of the population, the health management of older adults with comorbid COPD and diabetes is becoming increasingly complex, and the focus of their medical care is gradually shifting from acute hospital treatment to long-term community and home-based management (3, 4). In this context, the “hospital-to-home” transition period constitutes a particularly vulnerable and critical window in the patient’s health trajectory (5). Studies indicate that up to 30% of such patients experience adverse health outcomes during this phase, such as acute exacerbations, poor glycemic control, and unplanned rehospitalizations (6, 7).

Illness perception refers to the patient’s personal experience and cognitive representation of the disease, encompassing their understanding and emotional response regarding disease symptoms, causes, consequences, and controllability (8). In the context of COPD and diabetes comorbidity, patients often face a complex interplay of experiences such as dyspnea, fatigue, and various metabolic symptoms, making them highly susceptible to developing negative illness perceptions, such as feeling a loss of control, fearing deterioration, and losing confidence in treatment (9, 10). Concurrently, the World Health Organization’s concept of “intrinsic capacity” provides a novel framework for assessing the overall health reserve of older adults with comorbidities (11). It refers to the composite of an individual’s abilities across five domains: cognition, psychology, vitality, sensory, and locomotor (12). The physiological stress, environmental changes, and polypharmacy during the transition period can easily lead to a precipitous decline in the intrinsic capacity of older patients, thereby undermining the foundation for independent living and creating a vicious cycle of functional decline and deteriorating health status (13, 14).

Current research on transition management for patients with COPD or diabetes predominantly focuses on single diseases or individual outcomes (e.g., rehospitalization rates), lacking an in-depth exploration of the dynamic evolution and interactive influence between the two core psychological and functional constructs: “illness perception” and “intrinsic capacity.” However, according to the Common-Sense Model of Self-Regulation, an individual’s perception of their illness directly influences their coping behaviors and emotional adaptation, which in turn may accelerate or delay the decline of their physiological function (15). Conversely, a decline in intrinsic capacity (e.g., cognitive decline, physical frailty) may also impair the patient’s ability to process disease information and execute self-management, thereby exacerbating their perception of the disease as threatening (16, 17). This potentially bidirectional, dynamic interaction during the critical transition period is key to understanding the heterogeneity in patient outcomes, yet it has not been adequately illuminated by empirical research.

Therefore, this study aims to employ a longitudinal design to consecutively assess older adults with comorbid COPD and diabetes at hospital discharge, 1 month post-discharge, and 3 months post-discharge. By utilizing cross-lagged panel modeling and latent growth modeling, the study aims to: (1) delineate the changing trajectories of illness perception and intrinsic capacity during the transition period; and (2) clarify whether a mutual predictive relationship exists between them. The findings will provide a crucial theoretical basis and practical targets for constructing personalized transition care intervention programs centered on “optimizing perception and protecting function.”

## 2 Participants and methods

### 2.1 Participants

Using a convenience sampling method, older adults with COPD and diabetes who were treated in the five respiratory and critical care medicine wards and the respiratory outpatient clinic of the Sixth People’s Hospital of Nantong from April 2024 to September 2025 were selected as the survey participants.

*Inclusion criteria*: ① Aged ≥60 years; ② Meeting the diagnostic criteria for both COPD and T2DM according to the Chinese Guidelines for the Diagnosis and Management of Chronic Obstructive Pulmonary Disease (18) and the Chinese Guidelines for the Prevention and Treatment of Type 2 Diabetes (19); ③ Hospitalized for the current episode due to changes in condition such as acute exacerbation or poor glycemic control, and planning to be discharged home; ④ Clear consciousness with basic communication and comprehension abilities. Providing informed consent and voluntarily participating in the study.

*Exclusion criteria*: ① Comorbid with severe cardiac, hepatic, or renal insufficiency or other life-threatening severe diseases; ② Accompanied by severe cognitive impairment or mental illness, unable to cooperate in completing the assessments.

*Elimination and dropout criteria*: ① Missing key information in the baseline questionnaire ≥20%; ② Lost to follow-up or voluntarily withdrew at subsequent follow-up time points.

The primary analyses of this study employed structural equation modeling, specifically the Random Intercept Cross-Lagged Panel Model (RI-CLPM) and the Latent Growth Model (LGM). In the RI-CLPM, the observed variables comprised the total scores of illness perception and intrinsic capacity measured at three time points, yielding a total of six observed variables. According to classic methodological recommendations, the minimum sample size for structural equation modeling should be 10–20 times the number of observed variables, i.e., 60–120 cases, or no less than 200 cases (20). Given that the primary observed variables encompass multidimensional indicators of illness perception and intrinsic capacity, and considering the potential attrition inherent in a three-wave prospective cohort study, an anticipated dropout rate of 15–20% was assumed. Accordingly, the required initial sample size was calculated to be 240–250 cases. Ultimately, a total of 260 patients were enrolled and completed the baseline survey, meeting the sample size requirement. Referencing prior studies reporting a moderate effect size between illness perception and health outcomes (*r* ≈ 0.30) (21), as well as the common sample size convention for comparable longitudinal cross-lagged studies (typically 200–300 cases), the final analytical sample (*N* = 232) of the present study provides adequate statistical power to detect the significance of the core cross-lagged paths. The study protocol was reviewed and approved by the Ethics Committee of the Sixth People’s Hospital of Nantong (Approval No.: NTLYLL2024013). All participants provided written informed consent before participation.

### 2.2 Research instruments

#### 2.2.1 General information questionnaire

This questionnaire was self-designed by the researchers and consisted of two sections: (1) Demographic characteristics: including age, gender, education level, marital status, living situation, primary caregiver, and average monthly household income per capita. (2) Clinical characteristics: including COPD duration, Global Initiative for Chronic Obstructive Lung Disease (GOLD) classification, diabetes duration, most recent glycated hemoglobin (HbA1c) value, number and types of comorbidities, and number and types of medications.

#### 2.2.2 Assessment of illness perception

Illness perception was assessed using the Chinese version of the Brief Illness Perception Questionnaire (BIPQ) (22). This scale is used to evaluate patients’ cognitive and emotional representations of their illness. It consists of 8 items assessing the following dimensions: the consequences of the illness, timeline, personal control, treatment control, identity (symptom representation), concern, illness coherence (understanding), and emotional representation. Each item is scored on a 0–10 scale, with items 3, 4, and 7 being reverse-scored. The scores of all 8 items are summed to yield a total score ranging from 0 to 80. A higher total score indicates a stronger perception of illness threat, representing a more negative illness perception. This scale is widely used in patients with chronic diseases and demonstrates good reliability and validity. In this study, the Cronbach’s alpha coefficient for this scale was 0.832.

#### 2.2.3 Assessment of intrinsic capacity

Based on the World Health Organization’s framework of intrinsic capacity, assessment was conducted across five domains: cognition, psychology, vitality, sensory, and locomotor. Each domain was defined as a binary variable (normal = 1 point, impaired = 0 points). The total score ranged from 0 to 5, with a higher score indicating better overall intrinsic capacity status. The specific assessment tools and criteria were as follows: ① Cognitive function was assessed using the Mini-Cog. A total score ≥ 4 was defined as normal cognitive function, coded as 1 point (23). This cutoff value has demonstrated good sensitivity and specificity in older adults with chronic diseases and has been validated in multiple cognitive screening studies targeting patients with COPD and diabetes mellitus (24). The Mini-Cog is particularly suitable for rapid cognitive assessment of older patients with multimorbidity in busy clinical settings due to its ease of administration, short administration time, and minimal influence from language and educational level; ② Psychology: Assessed using the 2-item version of the Patient Health Questionnaire (PHQ-2) for depressive symptoms. The total score ranges from 0–6; a score > 3 was defined as impairment in the psychological domain (25); ③ Vitality: Assessed using the Mini Nutritional Assessment-Short Form (MNA-SF). The total score ranges from 0 to 14, with a cutoff of ≤ 11 indicating impaired vitality (malnutrition risk). The MNA-SF has been widely validated for nutritional screening in older patients with COPD and diabetes mellitus, and its cutoff demonstrates good sensitivity and specificity in identifying malnutrition risk (26). ④ Sensory: Assessed via patient self-report. Impairment in the sensory domain was defined if vision or hearing was self-rated as “poor” or “very poor,” or if the patient reported that vision or hearing problems affected daily life. ⑤ Locomotor: Assessed via the 6-meter walk test to evaluate gait speed. Participants walked 6 meters at their usual pace, the time was recorded, and gait speed (m/s) was calculated. According to the Asian Working Group for Sarcopenia consensus, a gait speed < 1.0 m/s was defined as impairment in the locomotor domain.

In this study, the pairwise correlation coefficients among the five domains at baseline ranged from 0.18 to 0.39, with a mean correlation of 0.28, indicating that the domains share a certain amount of common variance and that the total score can reflect a meaningful overarching construct. Multiple longitudinal studies based on the WHO framework have adopted the total intrinsic capacity score for cross-time comparisons, confirming its ability to sensitively capture trajectories of functional decline in older populations and demonstrating acceptable longitudinal measurement invariance (27, 28). As a simple and intuitive clinical screening indicator, the total score facilitates healthcare professionals in rapidly identifying individuals at high risk of functional decline, supporting its translational value. Therefore, the use of the total intrinsic capacity score for longitudinal comparisons in this study is both justified and feasible.

### 2.3 Data collection methods

Data for this study were collected by researchers who had undergone standardized training.

#### 2.3.1 Baseline survey

The baseline survey (T0) was conducted within 24–48 h prior to the patient’s discharge, inside the respiratory ward. Using a one-on-one, face-to-face interview format, the General Information Questionnaire and the Illness Perception Questionnaire were administered. Components of the intrinsic capacity assessment feasible at the bedside were also completed. The 6-meter walk test was performed under safe conditions in the ward corridor.

#### 2.3.2 Follow-up surveys and rationale for time points

All follow-up assessments were conducted via scheduled outpatient visits. To accurately track the dynamic changes in core variables during the high-risk hospital-to-home transition period, this study established three measurement time points: (1) T0 (Pre-discharge): To capture the patient’s baseline state immediately following acute medical intervention and just before leaving the supportive hospital environment, serving as the reference for evaluating subsequent changes. (2) T1 (1 month post-discharge): This stage represents a high-incidence window for adverse transition events (e.g., acute exacerbation, poor glycemic control), where patients face the greatest challenges in self-management. Follow-up at this time is most sensitive for reflecting their early psychological and functional adaptation processes. (3) T2 (3 months post-discharge): By this time, patients typically enter a phase of relatively stable home-based management. Assessment at this point reflects their stabilized illness cognition and longer-term functional level, representing a critical endpoint for evaluating transition outcomes. Follow-ups at T1 and T2 were conducted when patients returned for their scheduled outpatient review. Researchers made appointments in advance and completed all questionnaires and tests (identical in content to T0) in a private, quiet room. This approach ensured standardized execution of objective assessments like the walk test and guaranteed the quality and completeness of questionnaire responses.

#### 2.3.3 Quality control and sample attrition

All data collection adhered to the principles of informed consent and confidentiality. Participant enrollment for this study began in April 2024, and the baseline (T0) survey was completed by September 2025, with a total of 260 patients enrolled. T1 Follow-up: 18 patients did not return for their scheduled appointment (12 due to feeling well and not seeking review, 6 for other reasons). 242 patients completed the outpatient follow-up, yielding 242 valid questionnaires.

T2 Follow-up: Among those who completed T1, an additional 10 patients were lost to follow-up (6 did not return, 4 were re-hospitalized due to their condition). 232 patients completed the outpatient follow-up, yielding 232 valid questionnaires. The overall follow-up completion rate for all three measurements was 89.2% (232/260) (see Supplementary File 1). The missing data mechanism was assumed to be missing at Random (MAR), meaning that conditional on the observed baseline variables, the probability of missingness is unrelated to the unobserved outcome variables. Given the low overall attrition rate (10.8%) and the absence of statistically significant differences in key baseline characteristics between completers and dropouts, this study adopted Complete Case Analysis (CCA) as the primary analytical approach. To ensure the robustness of the findings, Multiple Imputation (MI) was also employed in sensitivity analyses, generating 20 imputed datasets based on chained equations, and the pooled results were consistent with those from CCA. To evaluate whether attrition introduced selection bias, compared key baseline characteristics between participants who completed all three follow-ups (*n* = 232) and those who missed at least one follow-up (*n* = 28). The results showed no statistically significant differences between the two groups in age (*t* = 0.87, *p* = 0.386), sex (χ^2^ = 0.24, *p* = 0.622), COPD severity (GOLD classification) (χ^2^ = 1.53, *p* = 0.675), baseline total illness perception score (*t* = 1.02, *p* = 0.308), or baseline total intrinsic capacity score (*t* = 0.76, *p* = 0.448), suggesting that attrition bias had a negligible impact on the study findings.

### 2.4 Statistical analysis

Data analysis was performed using R software (version 4.3.0), with a two-sided significance level set at *α* = 0.05. For descriptive analyses, continuous variables conforming to a normal distribution were presented as mean ± standard deviation, those not normally distributed were presented as median (interquartile range), and categorical variables were presented as frequency (percentage). Normality of illness perception scores at each time point was assessed using the Shapiro–Wilk test, and sphericity was evaluated using Mauchly’s test; if the sphericity assumption was violated, degrees of freedom were adjusted using the Greenhouse–Geisser correction. For structural equation modeling, multivariate normality was examined using Mardia’s test, and robust maximum likelihood estimation (MLR) was employed to mitigate the impact of non-normality on parameter estimates.

To investigate the dynamic relationship between illness perception and intrinsic capacity during the transition period (T0, T1, T2), two complementary models were constructed: Random Intercept Cross-Lagged Panel Model (RI-CLPM): Implemented using the lavaanpackage. After controlling for stable individual traits (random intercepts), this model examined the cross-lagged associations between illness perception and intrinsic capacity at adjacent time points (e.g., T0 illness perception → T1 intrinsic capacity), capturing within-individual dynamic interactions.

Latent Growth Model (LGM): Implemented using the lcmmpackage. Unconditional linear growth models were first fitted separately for illness perception and intrinsic capacity. Subsequently, a parallel process model was used to examine the associations between their initial levels and change slopes, exploring the covariation of their developmental trajectories at the overall trend level. Missing data were handled using full information maximum likelihood (FIML), and parameters were estimated using robust maximum likelihood (MLR). Model fit was evaluated against the following acceptable thresholds: CFI > 0.90, RMSEA < 0.08, and SRMR < 0.08.

To further explore the heterogeneity of the dynamic relationship between illness perception and intrinsic capacity across different patient subgroups, three potential moderators were prespecified based on prior clinical knowledge and literature evidence: COPD severity, baseline vitality status, and baseline cognitive function. Multi-group structural equation modeling was used to test differences in cross-lagged path coefficients between groups, with statistical significance evaluated using the Wald χ^2^ test. Given that these analyses were exploratory in nature—aimed at generating hypotheses for future research rather than testing prespecified hypotheses—no strict adjustment for multiple testing (e.g., Bonferroni correction) was applied; instead, raw *p*-values and effect sizes were reported to allow readers to assess the reliability of the findings.

The total intrinsic capacity score was derived by summing five dichotomous domains, yielding a range of 0–5, which qualifies as an ordinal variable. In this study, it was treated as a continuous variable in the LGM based on the following considerations: (1) Relatively wide range: The 0–5 scale comprises six levels, and in a longitudinal design with three time points, it can generate diverse patterns of change sufficient to support the estimation of linear growth parameters. (2) Approximately normal distribution: In this study, the absolute skewness values of the total intrinsic capacity score at all three time points were less than 1.0 (range −0.42 to 0.38), and the absolute kurtosis values were less than 1.5, approximating a normal distribution; thus, using a linear LGM would not introduce substantial estimation bias. (3) Prior precedent: Multiple longitudinal studies based on the WHO intrinsic capacity framework have employed linear LGM or linear mixed models to handle similar total score data, and these approaches have been accepted by mainstream journals (29). (4) Robust maximum likelihood estimation: The MLR estimator used in this study is robust to non-normal data, providing reliable standard errors and model fit indices. Nevertheless, the potential limitations of this approach are discussed in the limitations section.

## 3 Results

### 3.1 Baseline characteristics of the participants

Table 1 presents the baseline sociodemographic, disease-related clinical characteristics, and functional health status of the 232 participants who completed all three follow-up assessments. The mean age of the participants was 68.5 ± 5.2 years, with a range of 60 to 85 years.

**Table 1:** Baseline characteristics of the participants (*N* = 232).

Characteristic	Value (*n*)	Proportion (%)
Sociodemographic data		
Age (years, x̄ ± s)	68.5 ± 5.2	
Age group [*n* (%)]		
60–74 years	178	76.7
≥75 years	54	23.3
Gender [*n* (%)]		
Male	133	57.3
Female	99	42.7
Education level [*n* (%)]		
Primary school or below	102	44.0
Junior high school	76	32.7
Senior high school/Vocational school	42	18.1
College degree or above	12	5.2
Living Arrangement [*n* (%)]		
Living with spouse/children	197	84.9
Living alone	35	15.1
Primary medical insurance [*n* (%)]		
Urban employee basic medical insurance	151	65.1
Urban and rural resident basic medical insurance	81	34.9
Disease-related clinical characteristics		
COPD duration [years, M (IQR)]	8.0 (5.0, 12.0)	
COPD GOLD classification [*n* (%)]		
GOLD 2	100	43.1
GOLD 3	90	38.8
GOLD 4	42	18.1
Diabetes duration [years, M (IQR)]	8.0 (4.0, 12.0)	
Most recent HbA1c (%, x̄ ± s)	7.8 ± 1.2	
Charlson comorbidity index (CCI) [score, M (IQR)]	2 (1, 3)	
Current hospitalization length [days, M (IQR)]	6.0 (4.3, 11.0)	
Readmissions in the past year [*n* (%)]		
0 times	167	72.0
≥1 time	65	28.0
Functional and health status (intrinsic capacity domains)		
Intrinsic capacity total score [score, M (IQR)]	3 (2, 4)	
Domain impairment [*n* (%)]		
Cognitive impairment (Mini-Cog)	51	22.0
Psychological impairment (PHQ-2 > 3)	60	25.9
Vitality impairment (MNA-SF ≤ 11)	74	31.9
Sensory impairment (Self-reported)	126	54.3
Locomotor impairment (Gait Speed < 1.0 m/s)	112	48.3

COPD, Chronic Obstructive Pulmonary Disease; GOLD, Global Initiative for Chronic Obstructive Lung Disease; HbA1c, Glycated Hemoglobin; CCI, Charlson Comorbidity Index; M, Median; IQR, Interquartile Range; Mini-Cog, Mini-Cognitive Assessment; PHQ-2, 2-item Patient Health Questionnaire; MNA-SF, Mini Nutritional Assessment-Short Form.

### 3.2 Correlation and descriptive analysis of illness perception and intrinsic capacity

**Figure 1:**
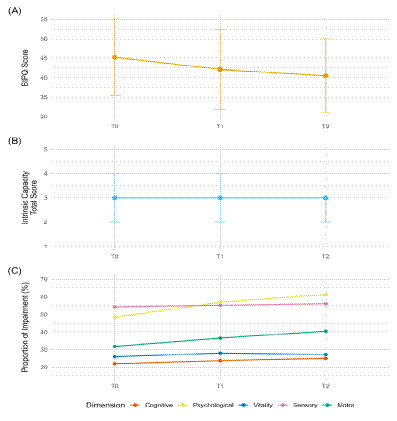
Trajectories of illness perception and intrinsic Capacityin older adults with COPD and diabetes during hospital-to-home transition. **(A)** Illness perception total score measured by Brief Illness Perception Questionnaire (BIPQ). **(B)** Intrinsic capacity total score. **(C)** Proportion of impairment across five intrinsic capacity dimensions. Error bars represent ±1 SD **(A,B)** or standard error of proportion **(C)**. T0, Predischarge; T1, 1 month after discharge; T2, 3 months after discharge.

The scores and changing trends of patients’ illness perception and intrinsic capacity across the three time points are shown in Table 2. Regarding illness perception, the total BIPQ score showed a gradual and significant decreasing trend over the three time points. Repeated measures analysis of variance revealed a significant main effect of time. *Post hoc* pairwise comparisons indicated that scores at both T1 and T2 were significantly lower than at T0, and the score at T2 was also significantly lower than at T1. This suggests that as patients transitioned from hospital to home, their overall perception of illness threat and negative cognition showed continuous improvement. Regarding intrinsic capacity, the total score differed statistically across the three time points, exhibiting a slow declining trend. The Friedman test with *post hoc* pairwise comparisons (Bonferroni corrected) showed that the total intrinsic capacity score at T2 was significantly lower than at T0. Further analysis of individual domains revealed that the impairment rates in the locomotor and vitality domains increased significantly during the transition period. Specifically, the locomotor impairment rate increased from 48.3% at T0 to 61.2% at T2, and the vitality impairment rate increased from 31.9% at T0 to 40.5% at T2. Changes in the impairment rates for the cognitive, psychological, and sensory domains were not statistically significant across the three time points (see Figure 1).

**Table 2:** Scores and comparisons of illness perception and intrinsic capacity across three time points (*N* = 232).

Variable	T0	T1	T2	Statistic	*p*-value
BIPQ					
Score (points, mean ± SD)	45.2 ± 9.8	42.1 ± 10.3^a^	40.5 ± 9.5^ab^	*F* = 15.73	<0.001
Intrinsic capacity total score					
Score [points, median (IQR)]	3 (2, 4)	3 (2, 4)	3 (2, 3)^a^	χ^2^ = 9.25	0.010
Intrinsic capacity domain impairment rate [*n* (%)]					
Cognitive impairment	51 (22.0)	55 (23.7)	58 (25.0)	0.72	0.697
Psychological impairment	60 (25.9)	65 (28.0)	63 (27.2)	0.31	0.856
Vitality impairment	74 (31.9)	85 (36.6)	94 (40.5)^a^	6.18	0.045
Sensory impairment	126 (54.3)	128 (55.2)	130 (56.0)	0.14	0.932
Locomotor impairment	112 (48.3)	132 (56.9)^a^	142 (61.2)^a^	10.56	0.005

BIPQ, Brief Illness Perception Questionnaire; SD, standard deviation; IQR, interquartile range. Comparison of the intrinsic capacity total score used the Friedman test (reporting χ^2^ value). Comparison of domain impairment rates used Cochran’s Q test (reporting Q value). ^a^Significantly different from T0, *p* < 0.05 (Bonferroni corrected). ^b^Significantly different from T1, *p* < 0.05 (Bonferroni corrected). Bolded *p*-values indicate statistical significance.

Correlation analysis further revealed the associations between illness perception and intrinsic capacity both within and across time points. As shown in Table 3, within each time point, illness perception and intrinsic capacity were significantly negatively correlated (T0: *r* = −0.42, *p* < 0.001; T1: *r* = −0.51, *p* < 0.001; T2: *r* = −0.45, *p* < 0.001), indicating that patients with more negative illness perception had worse intrinsic capacity. Cross-temporal correlations showed moderate to high stability of autocorrelations for each variable. Furthermore, illness perception at earlier time points was generally significantly negatively correlated with intrinsic capacity at subsequent time points, providing preliminary evidence for a potential dynamic interaction between the two constructs.

**Table 3:** Correlation matrix of illness perception and intrinsic capacity across three time points (*N* = 232).

Variable	1	2	3	4	5	6
1. BIPQ_T0	1					
2. BIPQ_T1	0.55^***^	1				
3. BIPQ_T2	0.48^***^	0.58^***^	1			
4. IC_T0	−0.42^***^	−0.35^***^	−0.30^**^	1		
5. IC_T1	−0.38^***^	−0.51^***^	−0.34^***^	0.48^***^	1	
6. IC_T2	−0.29^**^	−0.33^***^	−0.45^***^	0.35^***^	0.52^***^	1

BIPQ, Brief Illness Perception Questionnaire total score; IC, Intrinsic Capacity total score; T0, Pre-discharge; T1, 1 month post-discharge; T2, 3 months post-discharge. Spearman correlation coefficients are reported. *p < 0.05, *p* < 0.01, *p* < 0.001.

### 3.3 Analysis of the dynamic interplay between illness perception and intrinsic capacity

To delve into the dynamic interaction between illness perception and intrinsic capacity during the “hospital-to-home” transition, this study constructed a RI-CLPM and a LGM for analysis. The Shapiro–Wilk test showed that illness perception scores were approximately normally distributed at all three time points (T0: *W* = 0.98, *p* = 0.124; T1: *W* = 0.97, *p* = 0.086; T2: *W* = 0.98, *p* = 0.153). Mauchly’s test of sphericity was not significant (*W* = 0.95, *p* = 0.062), indicating that the sphericity assumption was satisfied. For the structural equation model, Mardia’s test indicated a slight deviation from multivariate normality (skewness *p* < 0.05, kurtosis *p* = 0.078); consequently, MLR estimation was employed to obtain robust standard errors and model fit indices.

#### 3.3.1 Model fit and trajectory analysis

**Figure 2:**
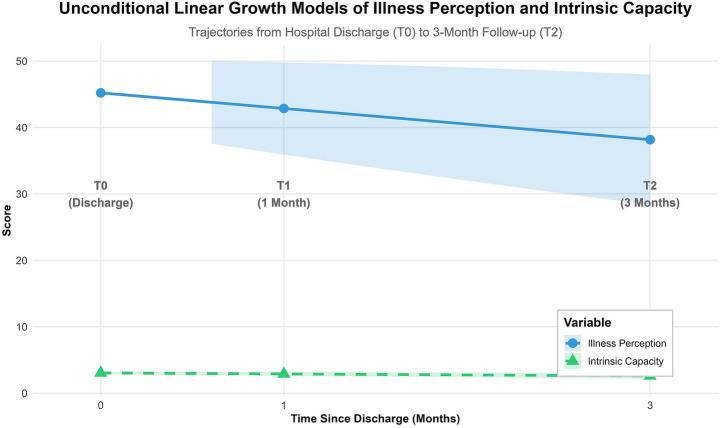
Unconditional linear growth models of illness perception and intrinsic capacity.

First, the goodness-of-fit indices for the RI-CLPM and the LGMs were examined, as shown in Table 4. The fit indices for the RI-CLPM indicated an acceptable model fit: χ^2^/df = 2.85, CFI = 0.94, TLI = 0.92, RMSEA = 0.07, SRMR = 0.05. The unconditional linear growth model for illness perception demonstrated a good fit (CFI = 0.98, TLI = 0.97, RMSEA = 0.04, SRMR = 0.03). Model estimation revealed a mean initial level (intercept) of 45.21 (*p* < 0.001) for illness perception, with significant between-individual variance (variance = 68.34, *p* < 0.001). The mean linear change slope was −2.35 (*p* < 0.001), indicating an overall improving trend, and the rate of improvement varied significantly between individuals (variance = 5.67, *p* = 0.012). The unconditional linear growth model for intrinsic capacity also demonstrated a good fit (CFI = 0.96, TLI = 0.95, RMSEA = 0.05, SRMR = 0.04). Its mean initial level was 3.05 (*p* < 0.001), with significant between-individual variance (variance = 0.89, *p* < 0.001). The mean change slope was −0.15 (*p* = 0.028), indicating a slight but significant linear declining trend, and the rate of decline varied significantly between individuals (variance = 0.08, *p* = 0.046) (see Figure 2 for details).

**Table 4:** Goodness-of-fit indices for the models.

Model	χ^2^/df	CFI	TLI	RMSEA	SRMR
Random-intercept cross-lagged panel model (RI-CLPM)	2.85	0.94	0.92	0.07	0.05
Unconditional linear growth model (illness perception)	-	0.98	0.97	0.04	0.03
Unconditional linear growth model (intrinsic capacity)	-	0.96	0.95	0.05	0.04

CFI, Comparative Fit Index; TLI, Tucker-Lewis Index; RMSEA, Root Mean Square Error of Approximation; SRMR, Standardized Root Mean Square Residual.

#### 3.3.2 Cross-lagged path analysis

**Figure 3:**
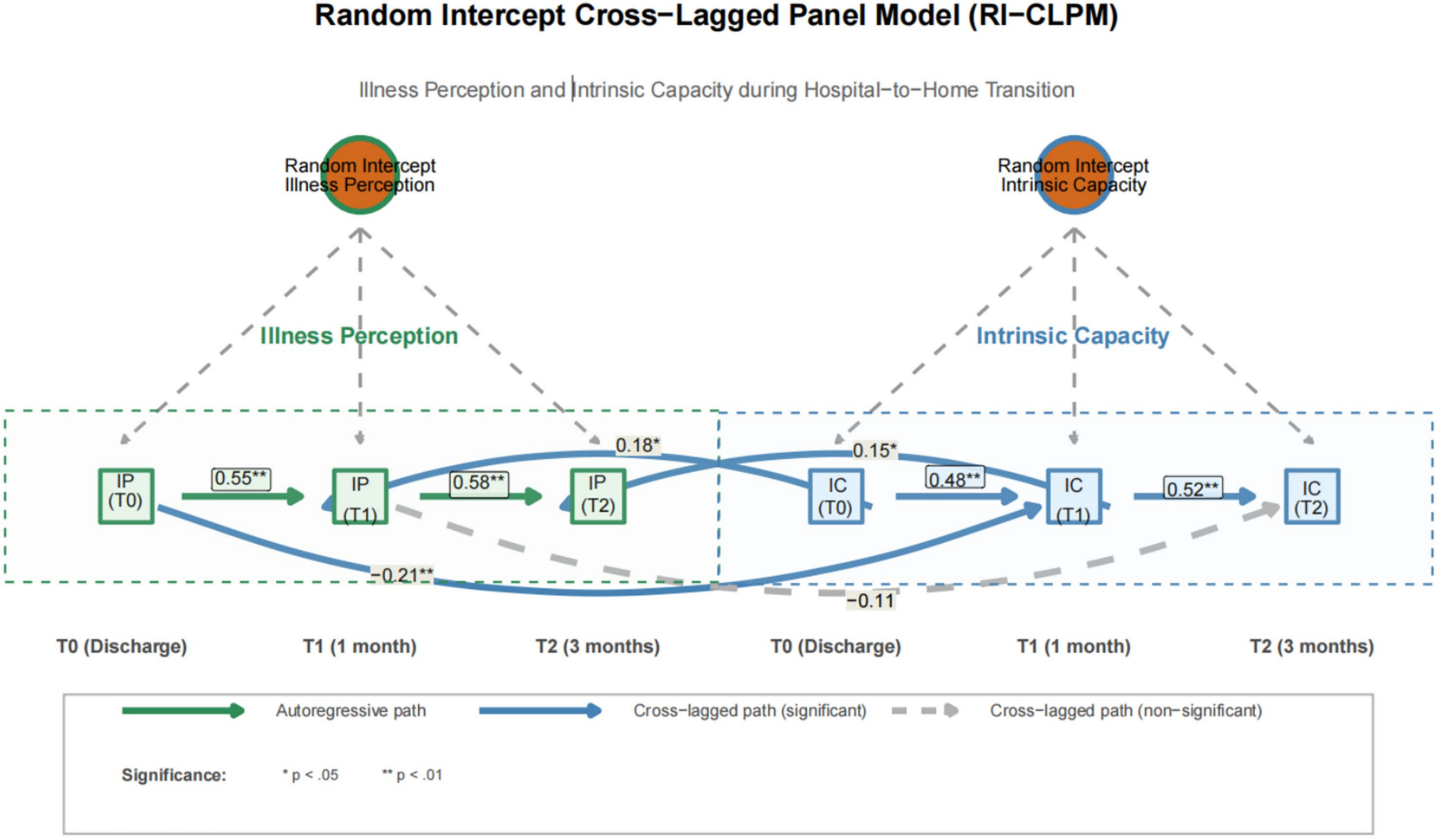
Diagram of the random-intercept cross-lagged panel model.

After controlling for stable individual trait factors, the random intercept cross-lagged panel model revealed dynamic mutual associations between illness perception and intrinsic capacity during the “hospital-to-home” transition period. The detailed results of the path analysis are summarized in Table 5. During the early transition phase (T0 to T1), there was a significant bidirectional negative association between illness perception and intrinsic capacity. Specifically, patients with more negative illness perception at discharge (T0) exhibited poorer intrinsic capacity one month later (T1) (*β* = −0.21, *p* = 0.003). Conversely, patients with poorer intrinsic capacity at T0 also reported more negative illness perception at T1 (*β* = 0.18, *p* = 0.012). However, during the later transition phase (T1 to T2), this pattern of mutual association changed. No significant association was found between illness perception at T1 and intrinsic capacity at T2 (*β* = −0.11, *p* = 0.105), whereas poorer intrinsic capacity at T1 remained significantly associated with more negative illness perception at T2 (*β* = 0.15, *p* = 0.047) (see Figure 3 for details). These findings suggest that the negative link between intrinsic capacity and subsequent illness perception may be more sustained throughout the entire transition period, whereas the negative link between illness perception and intrinsic capacity was relatively stronger during the early phase.

**Table 5:** Standardized path coefficient estimates for the random-intercept cross-lagged panel model.

Path direction	Predictor	Outcome variable	Standardized estimate (*β*)	95% confidence interval	*p*-value
Autoregressive paths	Illness perception (T0)	→ Illness perception (T1)	0.55	0.40, 0.70	< 0.001
	Illness perception (T1)	→ Illness perception (T2)	0.58	0.44, 0.72	< 0.001
	Intrinsic capacity (T0)	→ Intrinsic capacity (T1)	0.48	0.35, 0.61	< 0.001
	Intrinsic capacity (T1)	→ Intrinsic capacity (T2)	0.52	0.40, 0.64	< 0.001
Cross-lagged paths (T0 → T1)	Illness perception (T0)	→ Intrinsic capacity (T1)	−0.21	−0.35, −0.07	0.003
	Intrinsic capacity (T0)	→ Illness perception (T1)	0.18	0.04, 0.32	0.012
Cross-lagged paths (T1 → T2)	Illness perception (T1)	→ Intrinsic capacity (T2)	−0.11	−0.24, 0.02	0.105
	Intrinsic capacity (T1)	→ Illness perception (T2)	0.15	0.00, 0.30	0.047

#### 3.3.3 Association analysis of change trajectories

To investigate the association between their long-term change trends, a parallel process model incorporating the dual trajectories of illness perception and intrinsic capacity was constructed. The model demonstrated a good fit (CFI = 0.95, TLI = 0.93, RMSEA = 0.06, SRMR = 0.05). The analysis revealed a high negative correlation between the initial level of illness perception and the initial level of intrinsic capacity (*r* = −0.46, *p* < 0.001), indicating that patients with more negative illness perception at discharge had worse baseline intrinsic capacity. More importantly, a significant positive correlation was found between the change slope of illness perception and the change slope of intrinsic capacity (*r* = 0.32, *p* = 0.008). As the mean slopes for both variables in the unconditional models were negative (illness perception: −2.35, indicating improvement; intrinsic capacity: −0.15, indicating decline), this positive correlation implies that during the 3-month transition period, patients whose illness perception improved at a faster rate also experienced a relatively slower rate of decline in their intrinsic capacity. This strongly supports, at the level of overall developmental trajectories, a close and co-varying dynamic linkage between illness perception and intrinsic capacity (see Table 6).

**Table 6:** Correlation analysis of latent growth factors in the parallel process growth model.

Growth factor 1	Growth factor 2	Correlation coefficient (*r*)	*p*-value	Interpretation
Illness perception – initial level	Intrinsic capacity – initial level	−0.46	< 0.001	Patients with more negative illness perception at discharge had worse baseline intrinsic capacity.
Illness perception – change slope	Intrinsic capacity – change slope	0.32	0.008	Patients with a faster improvement rate in illness perception had a slower decline rate in intrinsic capacity.

The initial level refers to the intercept, representing the baseline value at discharge (T0). The change slope represents the linear rate of change from T0 to T2. All correlation coefficients are standardized estimates.

#### 3.3.4 Exploratory subgroup analysis

An exploratory subgroup analysis was conducted among the 232 patients with COPD and multimorbidity to examine whether the dynamic relationship between IP and IC varied according to patients’ baseline characteristics. This analysis involved a total of 3 prespecified moderators × 2 cross-lagged paths = 6 between-group comparisons. The sample sizes for each subgroup were as follows: COPD severity: mild-to-moderate (GOLD stage 2, *n* = 100) versus severe (GOLD stages 3–4, *n* = 132); baseline vitality status: normal vitality (MNA-SF > 11, *n* = 158) versus impaired vitality (MNA-SF ≤ 11, *n* = 74); baseline cognitive function: normal cognition (Mini-Cog ≥ 4, *n* = 181) versus impaired cognition (Mini-Cog < 4, *n* = 51). Because this analysis was exploratory in nature, aimed at generating rather than testing hypotheses, we reported both the uncorrected raw *p*-values and the *p*-values adjusted using the Bonferroni correction (corrected threshold *α* = 0.05/6 ≈ 0.0083). The results showed that COPD severity and baseline vitality status were key factors moderating this relationship. First, in the stratification by COPD severity, patients with severe disease (GOLD stages 3–4) exhibited a stronger negative association. Specifically, in the severe group, the negative predictive effect of illness perception on intrinsic capacity was statistically significant (*β* = −0.28, *p* = 0.001; Bonferroni-corrected *p* = 0.006), whereas it was not significant in the mild-to-moderate group (*β* = −0.12, *p* = 0.215). The between-group difference was statistically significant (Wald χ^2^ = 4.12, *p* = 0.042; corrected *p* = 0.252). Second, baseline vitality status showed a similar moderating trend. In the subgroup with impaired vitality (MNA-SF ≤ 11), the negative effect of illness perception on intrinsic capacity was stronger (*β* = −0.32, *p* = 0.002; corrected *p* = 0.012), whereas the path coefficient in the normal vitality group was weaker and not significant (*β* = −0.13, *p* = 0.954). The difference between the two groups was significant at the raw level (Wald χ^2^ = 3.89, *p* = 0.049) but did not reach statistical significance after Bonferroni correction (corrected *p* = 0.294), suggesting that this moderating effect warrants further validation in larger samples.

**Figure 4:**
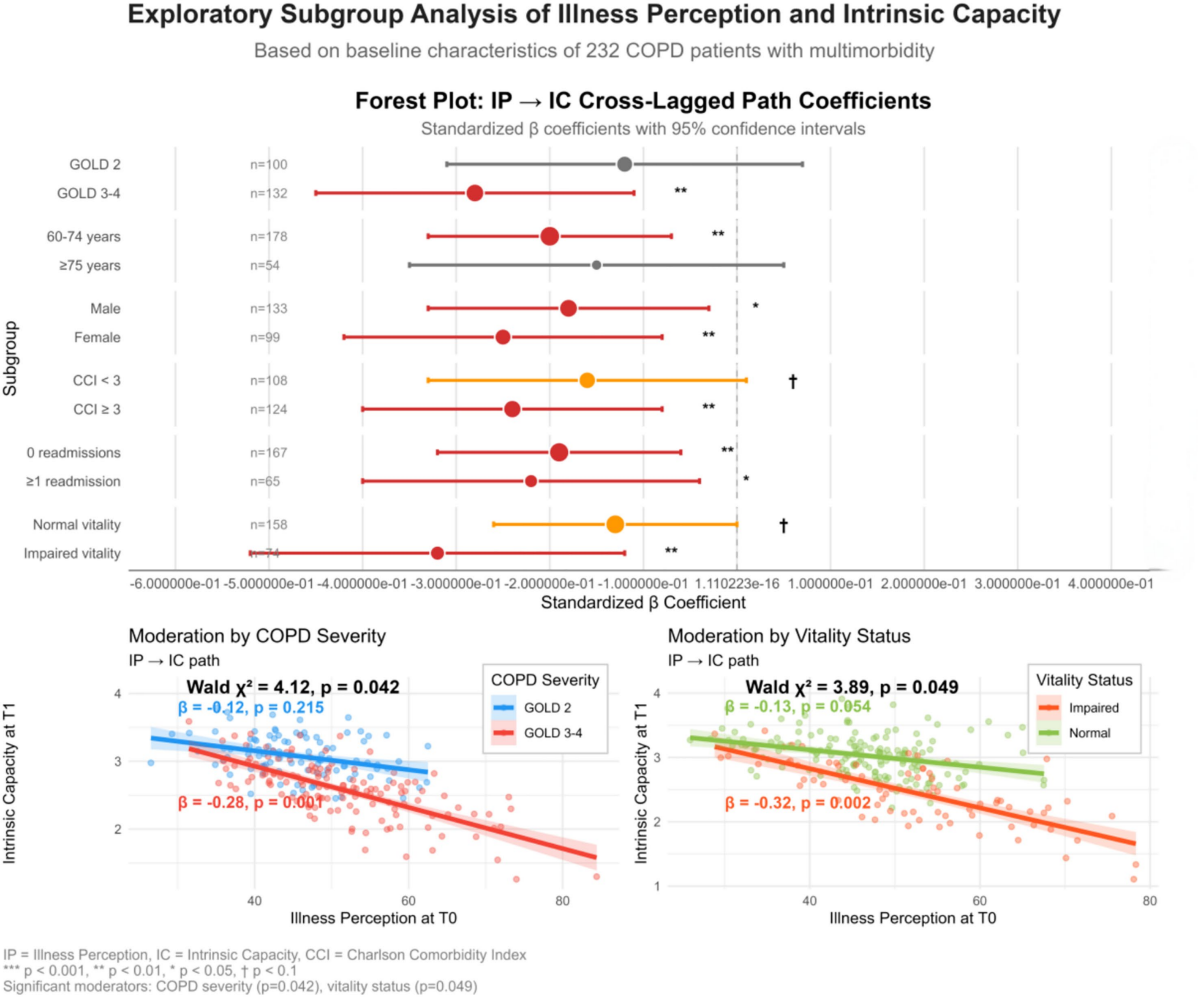
Exploratory subgroup analysis of illness perception and intrinsic capacity.

Furthermore, analysis of the reverse path (IC → IP) revealed that the negative association between intrinsic capacity and subsequent illness perception was more pronounced in the group with normal baseline cognition. The cognitively normal group exhibited a significant path coefficient (*β* = 0.20, *p* < 0.001; corrected *p* < 0.006), whereas no such association was found in the cognitively impaired group (*β* = 0.05, *p* = 0.623). The between-group difference was significant at the raw level (*p* = 0.045) but did not remain significant after correction (corrected *p* = 0.270). These results should be considered exploratory findings requiring confirmation in future confirmatory studies (see Figure 4).

## 4 Discussion

Through a 3-month longitudinal investigation of 232 older adults with comorbid COPD and diabetes, this study has preliminarily revealed the complex dynamic interaction between illness perception and intrinsic capacity during the “hospital-to-home” transition. At the overall level of the transition period, the findings indicate that patients’ illness perception showed a trend of improvement, while intrinsic capacity exhibited a slight decline. Furthermore, a significant association was found between their rates of change, suggesting that those with faster improvement in illness perception also experienced a slower rate of decline in intrinsic capacity. More importantly, after controlling for stable individual traits, cross-lagged model analysis demonstrated that in the early post-discharge phase (T0 to T1), a more negative illness perception and subsequently worse intrinsic capacity exhibited a significant mutual predictive relationship. Conversely, poorer intrinsic capacity also predicted subsequently more negative illness perception. As time progressed to the later phase (T1 to T2), this bidirectional pattern changed: the predictive effect of illness perception on intrinsic capacity weakened, while the negative influence of intrinsic capacity on illness perception appeared more sustained. Further exploratory analysis suggests that the aforementioned relationships are not homogeneous across all patients; the severity of COPD and the patients’ baseline nutritional and vitality status are potential important moderating factors.

These findings resonate with existing theoretical frameworks and empirical research while providing a new integrative perspective. Extensive studies support the predictive role of illness perception on health outcomes, with negative perceptions typically associated with poorer disease management behaviors and lower quality of life. For example, a 5-year prospective study of patients with multiple sclerosis found that negative components of illness perception, such as uncertainty about prognosis and symptoms and perceptions of impact on daily function, were associated with worse quality of life and higher levels of depression and anxiety (30). Research in patients with chronic obstructive pulmonary disease similarly shows that stronger illness identity, beliefs about a long illness timeline, perceptions of more severe consequences, and stronger emotional responses are all associated with lower quality of life, whereas perceiving higher personal control is associated with better quality of life (31). The present study not only confirmed the negative predictive power of illness perception on the comprehensive functional indicator of intrinsic capacity but also, through its temporal design, anchored this effect within the first month after returning home from the hospital. This period is characterized by drastic environmental changes and reconfiguration of support systems, when patients’ psychological vulnerability is highest. Their illness cognition may more readily activate physiological stress responses and influence health behaviors, thereby exerting a substantive erosive effect on limited functional reserves (32). This finding aligns with the theoretical expectations of the “cognitive stress model.” However, this study also revealed a reverse path that has been relatively overlooked in prior research: the significant predictive effect of intrinsic capacity on illness perception. This might be understood through the “somatic feedback” hypothesis: patients’ direct experience of somatic signals, such as declining daily activity capacity and increased fatigue, is continuously internalized and reshapes their judgment of disease severity and threat (32). Particularly in the later transition phase, as the impact of the acute hospitalization event fades, patients’ ongoing assessment of their own functional status may become the primary reality shaping their illness cognition. Therefore, the results of this study suggest that in the transitional care of older adults with multimorbidity, illness perception and intrinsic capacity may constitute a mutually influential, reinforcing dynamic system, rather than a simple unidirectional causal relationship.

Regarding the subgroup differences observed in this study, particularly the moderating effects of COPD severity and vitality status, the underlying mechanisms may involve multi-level interactions. The stronger association observed in patients with severe COPD may be related to the vulnerability of their physiological system. In these patients, respiratory functional reserve is severely compromised and operates at the edge of compensation. Any additional physiological or psychological stress (such as anxiety or hyperventilation triggered by negative cognitions) may more readily breach the threshold of their internal homeostasis, thereby becoming associated with more pronounced functional decline (33). In brief, the more severe the disease, the greater the patient’s physical vulnerability, and the stronger the association between psychological burden and functional decline. As for the moderating effect of baseline vitality status, it may reveal the buffering role of physiological reserve in the mind–body relationship. Adequate nutritional and metabolic status forms the foundation for maintaining muscle strength, immune function, and neural regulation. Sufficient physiological reserve may enhance an individual’s resilience in coping with psychological stress (34). Conversely, in patients at risk of malnutrition, whose physiological system is already in a state of depletion or compensation, the capacity to cope with additional challenges is diminished. This may render the psychological stress accompanying negative illness perception more likely to become associated with functional decline, forming a potential chain reaction (35, 36). Furthermore, the moderating effect of cognitive function on the intrinsic capacity-to-illness perception pathway suggests the importance of cognitive integrity. Intact cognitive function may be a prerequisite for patients to engage in accurate self-monitoring, reasonable attribution, and effective implementation of coping strategies. Cognitive impairment may weaken patients’ ability to adjust their illness cognition based on changes in their own functional status, thereby disrupting the effectiveness of this feedback loop.

Based on the above discussion, the results of this study carry multiple implications for clinical practice and future research. At the practical level, it firstly underscores the urgency for proactive screening and early intervention during the “hospital-to-home” transition, especially within the first month post-discharge. Clinicians should concurrently assess both the illness perception and the various domains of intrinsic capacity in patients, identifying those at high risk for both highly negative cognition and functional decline. Secondly, interventions need to be integrated and individualized. For illness perception, principles from illness perception therapy can be drawn upon, such as providing clear disease information, helping patients reframe their interpretation of symptoms, and setting realistic functional goals, to foster a more adaptive and controllable cognitive pattern. For intrinsic capacity, diversified support centered on functional maintenance should be implemented, including personalized pulmonary rehabilitation and physical training, nutritional optimization, correction of sensory impairments, and encouragement of social participation. A more innovative approach is to explore “mind–body integrated” intervention models. For example, cognitive-behavioral techniques could be organically incorporated into standard pulmonary rehabilitation training, guiding patients to attribute functional gains achieved during training to their own efforts and maintained abilities, thereby simultaneously enhancing their self-efficacy and improving illness perception.

Several limitations of this study should be acknowledged. First, this was a single-center convenience sampling study, with all participants recruited from one tertiary hospital in Nantong City, Jiangsu Province. The sample has certain limitations in terms of geographic region, hospital level, and socioeconomic status, which may affect the generalizability of the findings to other regions, primary care facilities, or different patient populations. Second, sensory function (vision and hearing) was assessed solely through patient self-report without objective testing. Self-reported measures may be influenced by factors such as cognitive status and emotional state, potentially introducing bias in the identification of sensory impairment. Although self-report offers advantages in clinical feasibility, future studies should adopt standardized objective assessment tools (e.g., visual acuity charts, audiometers) to improve measurement precision. Third, the intrinsic capacity domains were dichotomized into “normal/impaired” and summed to create a total score. While this approach ensures consistency with the international framework and enhances clinical utility, it may reduce sensitivity to subtle functional changes due to information simplification, potentially rendering the estimated trends and effect sizes for intrinsic capacity conservative. Fourth, the total intrinsic capacity score (ranging from 0 to 5, with only six possible values) was treated as a continuous variable in the latent growth model. Although this approach is justifiable given the distributional properties and precedents in prior research, strictly speaking, the variable is ordinal in nature. Linear models may not fully capture its discrete characteristics, and ceiling or floor effects may occur near the boundaries (e.g., scores of 0 or 5), potentially affecting the precision of slope estimates. However, the median baseline intrinsic capacity score in this study was 3, and no marked extreme clustering was observed across the three time points, suggesting that boundary effects may be limited. Fifth, this study found that the total intrinsic capacity score declined by an average of 0.15 points (on a 0–5 scale) during follow-up, a seemingly small change. Currently, no unified minimal clinically important difference (MCID) has been established for the total intrinsic capacity score. Drawing on analogous functional assessment tools in geriatric medicine (e.g., the MCID for the Short Physical Performance Battery [SPPB] is approximately 0.5 points), the 0.15-point decline observed in this study, although modest in absolute magnitude, still carries clinical alert value when considering the cumulative effect (approximately 0.30 points over one year if the trend continues), the population-level impact, and the synergistic effect with worsening illness perception. Sixth, although the random intercept cross-lagged panel model controlled for time-invariant confounders by separating between-person traits, this study remains observational in design and cannot rule out the potential influence of unmeasured time-varying confounders (e.g., changes in social support, medication adjustments, acute exacerbation events) on the directional pathways. Therefore, the bidirectional associations identified in this study should be interpreted as dynamic correlations rather than definitive causal relationships. Future studies employing stricter causal inference methods, multi-center designs, standardized objective assessment tools, and more refined measurement indicators (e.g., raw continuous variables or Poisson LGM models) are needed to further validate and extend the present findings.

## 5 Conclusion

In summary, this longitudinal study suggests that in older adults with COPD and multimorbidity, illness perception and intrinsic capacity exhibit a dynamic interplay during the hospital-to-home transition. This interaction is characterized by a mutual predictive relationship in the early phase, with the influence of intrinsic capacity potentially becoming more sustained in the later phase. This relationship is moderated by the severity of the patient’s COPD and their baseline physiological reserve. These findings provide preliminary evidence for developing transition support strategies that are timely, targeted, and holistic. They underscore the potential importance of breaking the “cognitive-functional decline” cycle in improving the long-term outcomes of this vulnerable population.

## Data Availability

The original contributions presented in the study are included in the article/Supplementary material, further inquiries can be directed to the corresponding author.
