# Supplementary material for: The interaction between illness perception and intrinsic capacity during hospital-to-home transition in older adults with COPD and diabetes: a prospective cohort study
Source: Front Med (Lausanne). 2026 Jul 22;13:1896128. doi: 10.3389/fmed.2026.1896128 (PMC13437485; doi:10.3389/fmed.2026.1896128)
Supplement: Supplementary file 1 [file Image_1.pdf]

Supplementary File 1

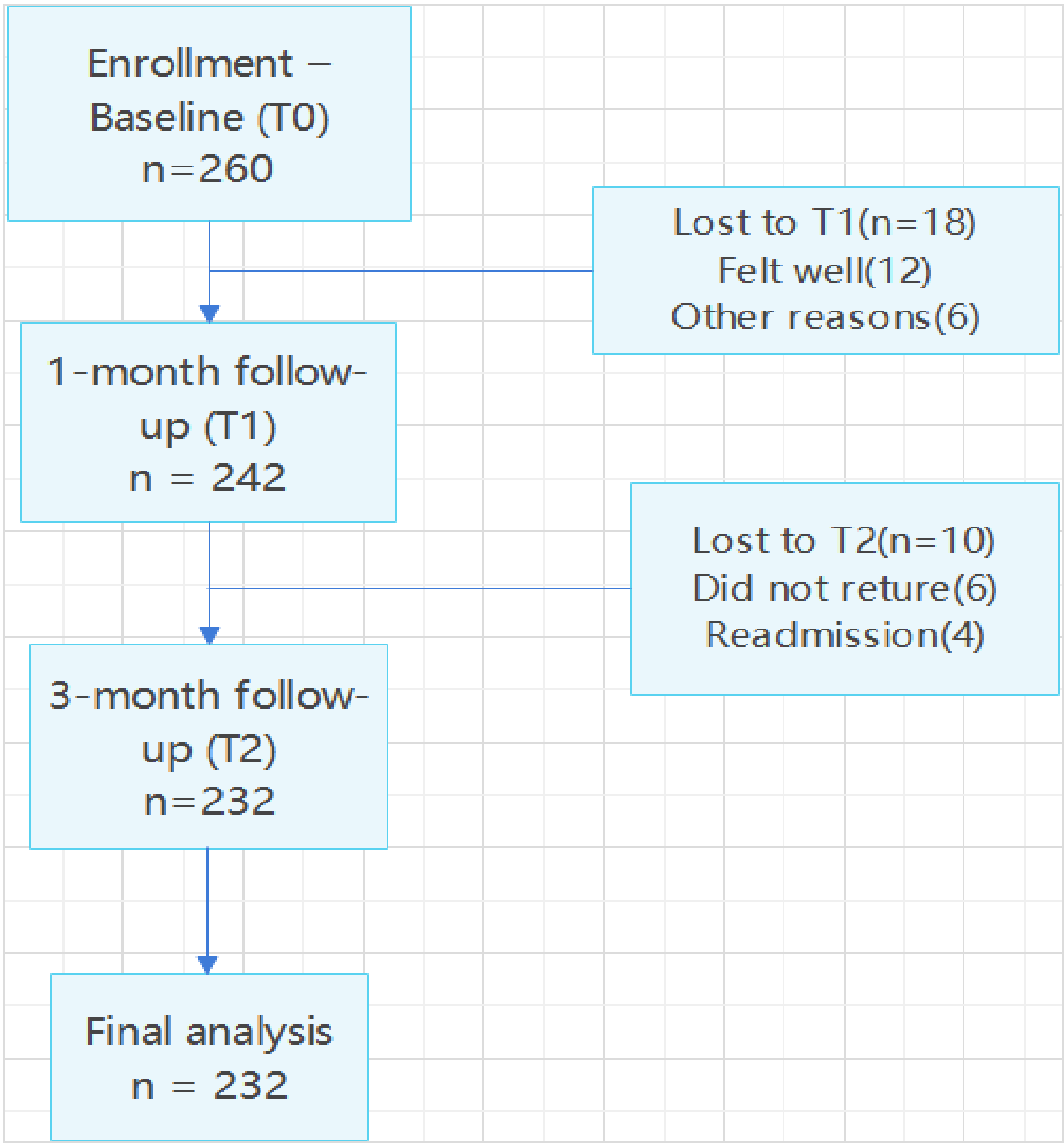

Supplementary Figure. Participant flow diagram.
